# Supplementary material for: The effect of using light emitting diodes and fluorescent lamps as different light sources in growth inhibition tests of green alga, diatom, and cyanobacteria
Source: PLoS One. 2021 Feb 19;16(2):e0247426. doi: 10.1371/journal.pone.0247426 (PMC7895407; doi:10.1371/journal.pone.0247426)
Supplement: Attachment — Submitted filename: Response to editor and reviewers.docx [file pone.0247426.s001.docx]

For editor,

Please ensure that your manuscript meets PLOS ONE's style requirements, including those for file naming.

We revised manuscript style. Moreover, this manuscript was checked by native check service.

Thank you for stating the following in the Financial Disclosure section:

"The authors received no specific funding for this work."

We note that one or more of the authors are employed by a commercial company: "Nippon Soda Co., Ltd.,"

Although the author is employed by Nippon Soda and receives an annual salary, the company did not provide financial assistance or funding, and only provided equipment and facilities. Nippon Soda did not play a role in the study design, data collection and analysis, decision to publish, or preparation of the manuscript.

Please also provide an updated Competing Interests Statement declaring this commercial affiliation along with any other relevant declarations relating to employment, consultancy, patents, products in development, or marketed products, etc.

The authors have declared that no competing interests exist.

Reviewer #1: Recommendation: Minor concerns
The manuscript entitled, “Effects of different lighting sources on the growth inhibition tests of green alga, diatom, and cyanobacteria” represents very interesting results, however, there are some minor concern regarding this study. If these concerns/queries are satisfied, it can be considered for publication.
Research title:
•The title of research “effects of different lighting sources” is very general, rephrase it.

We revised title:

The effect of using light emitting diodes and fluorescent lamps as different light sources in growth inhibition tests of green alga, diatom, and cyanobacteria

In abstract section:
•Please add an introductory line about why algal growth inhibition test is performed, discuss rationale behind the research.
We added sentence (LINE 13, 14):

Aquatic organisms have been used to investigate the safety of chemicals worldwide. One such assessment is an algal growth inhibition test.

In introduction section:
•Line 44, 45, please elaborate if LED or FL is better? This literature does not support or negate the research study.

We added sentence (LINE 48-53):

LEDs have also been used for illumination in greenhouse plants. Singh D et al. (2015) said “It is necessary to further investigate the not yet fully understood physiological processes mediating plant responses to LED light; however, economic analysis has clearly shown that LEDs can reduce the electricity cost, and investment (high capital cost) will be returned as profit in long-term operations in greenhouse industries” [5]. Reports about the effect of LEDs on plant growth have led to several studies comparing LEDs with FLs.

We added our thought in the conclusion: LEDs thus appear advantageous over FLs in algal growth inhibition tests.

•Line 51, “algal growth inhibition tests” are cultured or algae are cultured?
We revised sentence (LINE 59, 60):

Currently, algae in algal growth inhibition tests are commonly cultured using growth chambers with FLs.

•Line 57-58, “as no comparison test between LED and FL was conducted” please rephrase it with “has been conducted”.
We revised sentence (LINE 66).

•Please mention about the role of LED/FL illumination in algal growth inhibition tests.
We added sentence (LINE 67, 68):

Light is essential for the growth of algae, and light sources are therefore important for algal growth inhibition tests.

In methodology and results section:
•This section is fine, and methodology and results are in coherence with each other.
•However, the figures added could be of better resolution.
We revised figure resolution.

In discussion section:
•Please improve the discussion section, and make your research work more attractive.
We added sentence (Line 263-270).

Moreover, LEDs are being used increasingly in the commercial field where it is shown that the initial high capital cost of LEDs is returned as profit in certain sectors due to numerous advantages over FLs. Furthermore, LEDs do not contain mercury and have a low health impact due to low UV. LEDs have benefits in terms of decreased electricity cost and the reduced negative impact on the health of environmental organisms (including humans). In addition, they do not differ from conventional lighting sources when promoting growth of plants (including algae). LEDs thus appear advantageous over FLs in algal growth inhibition tests.

•Line 213, this information is not present in the cited reference 10. Please, check.
References:
We revised the reference on Line 213. We added a reference, therefore the reference number did not change.

•The references are not uniformly cited, e.g. reference 10, journal name is abbreviated while rest are not.

We revised reference style.

Reviewer #2: Comment and suggestion to authors:

PONE-D-20-28966
Titled: "Effects of different lighting sources on the growth inhibition tests of green alga, diatom, and cyanobacteria".

1) In this manuscript, the authors compared only between fluorescent lamp and light-emitting diode as the lighting sources, so it may not suitable to use the title “Effects of different lighting sources…..”. It would be better to improve the title to be more specific and clearer.
We revised title:

The effect of using light emitting diodes and fluorescent lamps as different light sources in growth inhibition tests of green alga, diatom, and cyanobacteria

2) This study employed only 1 species for green alga, 1 species for diatom and 1 species for cyanobacteria, why did each species can be the representative species to evaluate the effect of different lighting sources on the growth inhibition? This point should be clarified in the manuscript.

We added sentence (LINE 71-74).

We selected *P. subcapitata*, *N. pelliculosa,* and *A. flos-aquae* because these algae are used to evaluate the ecotoxicity of industrial chemicals and pesticides and are recommended in test guidelines.

3) Why did *Pseudokirchneriella subcapitata*, *Navicula pelliculosa,* and *Anabaena flos-aquae* is suitable for this study? Why don’t the authors used other species or more number of species for their experiment? This point should be clarified in the manuscript as well.
We added sentence (LINE 86-88).

*P. subcapitata*, *A. flos-aquae* and *N. pelliculosa* were used for this study because these algae are recommended in OECD test guideline No.201, OCSPP number 850.4500 and 850.4550.

We could not conduct algal growth inhibition tests with other species, because we cannot buy fluorescent lamp.

4) There are some spelling mistakes and grammatical error found in this manuscript, the author should pay more attention on this point and check the whole manuscript before re-submission.

This manuscript was checked by native check service before the resubmission.
