# Supplementary material for: The effect of using light emitting diodes and fluorescent lamps as different light sources in growth inhibition tests of green alga, diatom, and cyanobacteria
Source: PLoS One. 2021 Feb 19;16(2):e0247426. doi: 10.1371/journal.pone.0247426 (PMC7895407; doi:10.1371/journal.pone.0247426)
Supplement: Attachment — Submitted filename: Response_to_reviewers.docx [file pone.0247426.s002.docx]

Reviewer #2: Comment and suggestion to authors:

I appreciate your kind confirmation our manuscript during your busy time. We agree with you and revised our manuscript.

1) We revised LINE 86–89.

“*P. subcapitata*, *N. pelliculosa*, and *A. flos-aquae* are recommended in OECD test guideline No. 201 and OCSPP number 850.4500 and 850.4550 [6–8], and the test methods are internationally recognized. Moreover, the results of these species are required in registrations of pesticides or industrial chemicals. Therefore, these three algal species were used for this study.”

2) This manuscript was checked and revised by native speaker of English before the re-submission.
